# Two cyclic hexapeptides from *Penicillium* sp. FN070315 with antiangiogenic activities

**DOI:** 10.1371/journal.pone.0184339

**Published:** 2017-09-26

**Authors:** Jun-Pil Jang, Hye Jin Jung, Jang Mi Han, Narae Jung, Yonghyo Kim, Ho Jeong Kwon, Sung-Kyun Ko, Nak-Kyun Soung, Jae-Hyuk Jang, Jong Seog Ahn

**Affiliations:** 1 Anticancer Agent Research Center, Korea Research Institute of Bioscience and Biotechnology, Cheongju, Republic of Korea; 2 Deparment of BT-Convergent Pharmaceutical Engineering, Sun Moon University, Chungnam, Republic of Korea; 3 Department of Biotechnology, Yonsei University, Seoul, Republic of Korea; Medical College of Wisconsin, UNITED STATES

## Abstract

In the course of searching for angiogenesis inhibitors from microorganisms, two cyclic peptides, PF1171A (**1**) and PF1171C (**2**) were isolated from the soil fungus *Penicillium* sp. FN070315. In the present study, we investigated the antiangiogenic efficacy and associated mechanisms of **1** and **2** *in vitro* using human umbilical vein endothelial cells (HUVECs). Compounds **1** and **2** inhibited the proliferation of HUVECs at concentrations not exhibiting cytotoxicity. Moreover, **1** and **2** significantly suppressed vascular endothelial growth factor (VEGF)-induced migration, invasion, proliferation and tube formation of HUVECs as well as neovascularization of the chorioallantoic membrane in developing chick embryos. We also identified an association between the antiangiogenic activity of **1** and **2** and the downregulation of both the phosphorylation of VEGF receptor 2 and the expression of hypoxia inducible factor-1α at the protein level. Taken together, these results further suggest that compounds **1** and **2** will be promising angiogenesis inhibitors.

## Introduction

Natural products from microorganisms have provided various chemical templates for clinically useful lead compounds in the pharmaceutical industry [1, 2]. Particularly, fungi continue to be a rich source of biologically active secondary metabolites belonging to highly diverse structural classes, including alkaloids, macrolides, terpenoids, and peptides.^3–6^ These fungal metabolites have been reported to possess various biological properties such as antibacterial, antitumor and anti-inflammatory activities [3–6].

Angiogenesis, the growth of new blood vessels, is a complex process involving several steps including proliferation, migration and formation of capillary tubes in endothelial cells [7, 8]. Abnormal angiogenesis often occurs in pathological conditions such as cancer, rheumatoid arthritis, diabetic retinopathy and other chronic inflammatory diseases. The vascular endothelial growth factor (VEGF) family and related VEGF receptors (VEGFRs) have a central role in the modulation of pathological angiogenesis [9, 10]. VEGF has been shown to strongly induce cell migration, proliferation, and tube formation with a unique specificity for endothelial cells [11]. Additionally, VEGF is the key mediator of angiogenesis in cancer, in which it is upregulated by oncogene expression, a variety of growth factors and also hypoxia inducible factor (HIF) [12,13]. Based on these finding, VEGF signaling has been a target for the treatment of angiogenesis-related diseases including cancer. Previously reported fungal metabolites, such as epoxyquinols A [14] and B [15], azaspirene [16] and RK-95113 [17] have been evaluated for their anti-angiogenic activity. In the course of searching for secondary metabolites from microorganisms with biological activity, two cyclic peptides, PF1171A (**1**) and PF1171C (**2**) were isolated from the soil fungus *Penicillium* sp. FN070315. In this paper, we report the isolation and structural elucidation of **1** and **2** as well as demonstrate their antiangiogenic effect for the first time. Furthermore, molecular mechanisms involved in the antiangiogenic effect of **1** and **2** were elucidated.

## Materials and methods

### General experimental procedures

All solvents and reagents were of analytical grade and purchased from commercial sources. UV spectra and optical rotations were recorded on a BECKMAN DU^®^ 530 Life Science UV/Vis spectrophotometer and a HORIBA SEPA-300 high sensitive polarimeter, respectively. IR spectra were recorded on a HORIBA FT-720 IR spectrometer with a DuraSampl IR II ATR instrument. NMR spectra were recorded on a JEOL ECA-500 FT-NMR spectrometer at 500 MHz for ^1^H NMR and 125 MHz for ^13^C NMR. Chemical shifts were reported in ppm and referenced against the residual undeuterated solvent. Mass spectra were obtained on an AB Sciex Qtrap (ESIMS) and ABI3200, and HRESIMS was accomplished on a Waters Synapt GII. DAD-LC/MS analysis was performed using a Waters Alliance 2965 HPLC system, attached to a Waters 2996 PDA detector, with a Waters Xterra C_18_-column (5 *μ*m, 2.1 mm i.d. x 150 mm) that was connected to an AB Sciex Qtrap MS/MS system equipped with an ESI probe. Middle-pressure liquid chromatography (MPLC) was done with a Teledyne ISCO CombiFlash Companion. Preparative HPLC was performed using a Waters 600E pump system with Senshu Pak Pegasil ODS column (5 μm, 10 mm i.d. x 250 mm).

Endothelial growth medium-2 (EGM-2) was obtained from Lonza (Walkersville, MD, USA). Dulbecco’s modified Eagle’s medium (DMEM) and fetal bovine serum (FBS) were purchased from Invitrogen (Grand Island, NY, USA). Recombinant human vascular endothelial growth factor (VEGF), Matrigel, and Transwell chamber systems were obtained from Koma Biotech (Seoul, South Korea), BD Biosciences (San Jose, CA, USA), and Corning Costar (Acton, MA, USA), respectively. Anti-hypoxia inducible factor-1α (HIF-1α) antibody was purchased from BD Biosciences. Anti-phospho-VEGFR2, anti-VEGFR2, and anti-β-actin antibodies were purchased from Cell Signaling Technology (Beverly, MA, USA).

### Fungal material

Strain of a fungus, FN070315 was isolated from a soil sample collected from a small hill in KRIBB, Daejeon, Korea and identified based on the ribosomal RNA (rRNA) sequences and morphological evaluation. Approval or permission was not needed to collect samples. A GenBank search with the 26S rRNA gene of FN070315 indicated *Penicillium jensenii* (AY443470) and *Penicillium canescens* (AY484896) as the closest matches, with sequence identities of 100% and 99.98%, respectively. Therefore, the fungal strain FN070315 was identified and named as a *Penicillium* sp. FN070315 (deposited as KCTC1818P at the Korean Collection for Type Culture).

### Fermentation, extraction, and purification of secondary metabolites

*Penicillium* sp. FN070315 was grown on PD agar medium for 7 days and then inoculated into a 500-mL Erlenmeyer flask containing 75 mL of seed culture medium PD broth (24 g/L potato dextrose; BD Bioscience, San Jose, CA, USA). Incubation was carried out at 28°C for 3 days on a rotary shaker operating at 135 rpm. This seed medium (150 mL) was transferred to 8 L of the same production medium in a two 14-L jar fermentor. The fermentation was carried out at 28°C for 6 days with agitation at 165 r.p.m. and an air flow of 10 L/min. The culture broth (16 L) was filtered and extracted three times with an equal volume of EtOAc and the EtOAc layer was concentrated *in vacuo*. The EtOAc extract (1.3 g) was subjected to reversed-phase C_18_ flash column chromatography with a stepwise gradient of MeOH/H_2_O (from 20/80, 40/60, 60/40, 80/20 to 100/0; 700 mL for each step), to yield five fractions (fractions 1–5). The active fraction 3 (68.0 mg) eluted with MeOH/H_2_O (60/40) was purified by semi-preparative reverse phase HPLC with an isocratic solvent system of MeCN/H_2_O (50/50), to yield compound **1**(t_R_ 16.3 min, 6.5 mg) and **2** (t_R_ 23.5 min, 12.5 mg). The compouds **1** and **2** were dissolved in dimethyl sulfoxide (DMSO) at a concentration of 100 mM as a stock solution. In all kinds of cell-based assays performed in this study, the stock solution was further diluted with culture media for appropriate working doses. The negative control groups were treated with equal volumes of DMSO.

### Cell culture and hypoxic conditions

Early passages (4–8 passages) of human umbilical vein endothelial cells (HUVECs) and HepG2 (human hepatocarcinoma) cells were grown in EGM-2 and DMEM supplemented with 10% FBS, respectively. All cells were maintained at 37°C in a humidified 5% CO_2_ incubator. For hypoxic conditions, cells were incubated in a hypoxic chamber (Forma Scientific, Marietta, OH) under 5% CO_2_ and 1% O_2_ balanced with N_2_.

### Cell viability assay

HUVECs (3 × 10^3^ cells/well) were seeded in gelatin-coated 96-well culture plate and then treated with various concentrations of **1** and **2** for 72 h. Cell viability was measured with the 3-(4,5-dimethylthiazol-2-yl)-2,5-diphenyltetrazolium bromide (MTT) colorimetric assay (Sigma-Aldrich, Saint Louis, MO, USA).

### Trypan blue exclusion assay

HUVECs were seeded at a density of 1 × 10^5^ cells/well in gelatin-coated 12-well culture plate. Compounds **1** and **2** (25, 50 μM) were added to each well and the cells were incubated for up to 72 h. After 72 h, the cells were stained with trypan blue (Sigma-Aldrich) and counted with a hemocytometer. Cell viability was calculated as the number of viable cells divided by the total number of cells.

### Wound healing assay

The confluent monolayer HUVECs were scratched with a tip, and each well was washed with PBS to remove nonadherent cells. The cells were treated with **1** and **2** (25, 50 μM) in the presence of VEGF (30 ng/mL) and then incubated for up to 48 h. The perimeter of the area with a central cell-free gap was confirmed at the time intervals 0, 24, and 48 h under an optical microscope (Olympus, Center Valley, PA, USA).

### Chemoinvasion assay

The invasiveness of the HUVECs was investigated with a Transwell chamber system with polycarbonate filter inserts with a pore size of 8.0 μm. Briefly, the lower side of the filter was coated with 10 μL of gelatin (1 mg/mL) and the upper side was coated with 10 μL of Matrigel (3 mg/mL). Serum-starved HUVECs (8 × 10^4^ cells) were placed in the upper chamber of the filter and **1** and **2** (25, 50 μM) were added to the lower chamber in the presence of VEGF (30 ng/mL). The chamber was incubated at 37°C for 18 h, and then the cells were fixed with methanol and stained with hematoxylin/eosin. The total number of cells that invaded the lower chamber of the filter was counted with an optical microscope (Olympus) at a 100 × magnification.

### Adhesion assay

Serum-starved HUVECs (1.5 × 10^5^ cells) were treated with VEGF (30 ng/mL) in the presence or absence of **1** and **2** (25 and 50 μM) and then added in laminin-coated 24-well cuture plate. After incubation for 2 hrs, non-adherent cells were removed with gentle washing, and attached cells were counted under an optical microscope (Olympus).

### Capillary tube formation assay

Serum-starved HUVECs (8 × 10^4^ cells) were inoculated on a surface containing Matrigel (10 mg/mL) and incubated with **1** and **2** (25, 50 μM) for 6 h in the presence of VEGF (30 ng/mL). Morphological changes of the cells and tube formation were visualized under a microscope and photographed at a 100 × magnification (Olympus). Tube formation was quantified by counting the total number of branched tubes in randomly selected fields at a 100 × magnification.

### Chorioallantoic membrane (CAM) assay

Fertilized chick eggs were maintained in a humidified incubator at 37°C for 3 days. Approximately 6 mL of egg albumin were removed with a hypodermic needle, allowing the CAM and yolk sac to drop away from the shell membrane. After 2 days, the shell was punched out and peeled away. Thermanox coverslips (NUNC, Rochester, NY) loaded with vehicle alone, retinoic acid (RA), **1** or **2** were air-dried and applied to the CAM surface. Two days later, 2 mL of 10% fat emulsion (Greencross Co., Yongin, South Korea) were injected into the chorioallantois, and the CAM was observed under a microscope.

### Western blot analysis

Cell lysates were separated by sodium dodecyl sulfate–polyacrylamide gel electrophoresis (SDS-PAGE), and the separated proteins were transferred to poly vinylidene difluoride (PVDF) membranes (Millipore, Billerica, MA, USA) with standard electroblotting procedures. The blots were blocked and immunolabeled with primary antibodies against phospho-VEGFR2, VEGFR2, HIF-1α, and β-actin overnight at 4°C. Immunolabeling was detected with an enhanced chemiluminescence (ECL) kit (Bio-Rad, Hercules, CA, USA), according to the manufacturer’s instructions.

### RNA isolation and quantitative real-time PCR analysis

Total RNA content of cells was isolated using a RNA-spin^™^ Total RNA extraction kit according to the manufacturer's protocol (Intron Biotechnology, Korea). RNA concentrations were determined using a Qubit^®^ 2.0 Fluorometer RNA assay kit (Life Technologies, USA). cDNA was prepared using a Maxime RT Premix cDNA synthesis kit (Intron Biotechnology, Korea) according to the manufacturer's protocol. Quantitative PCR was performed using 0.9 μM each of forward and reverse primers (VEGFR-2; forward 5’- AGCGATGGCCTCTTCTGTAA -3’, reverse 5’- ACACGACTCCATGTTGGTCA -3’, GAPDH; forward 5’- GGTCTCCTCTGACTTCAACA -3’, reverse 5’- GTTGCTTAGCCAAATTCGTT -3’) with SYBR Green premix (Life technologies, USA) on a real-time PCR system (Bio-Rad CFX96, USA).

### Measurement of VEGF by enzyme-linked immunosorbent assay (ELISA)

The VEGF concentration in the media from the cells treated with **1** and **2** (25, 50 μM) was determined with a VEGF immunoassay kit (R&D Systems, Minneapolis, MN, USA) according to the manufacturer’s instructions. The results were expressed as the concentration of VEGF relative to the total amount of protein from each well.

### Statistical analysis

The results are expressed as the mean ± standard error (SE). Student’s *t* test was used to determine statistical significance between the control and test groups. A *P* value of < 0.05 was considered statistically significant.

## Results and discussion

### Isolation and identification of cyclic hexapeptides, PF1171A (1) and PF1171C (2)

**Fig 1:**
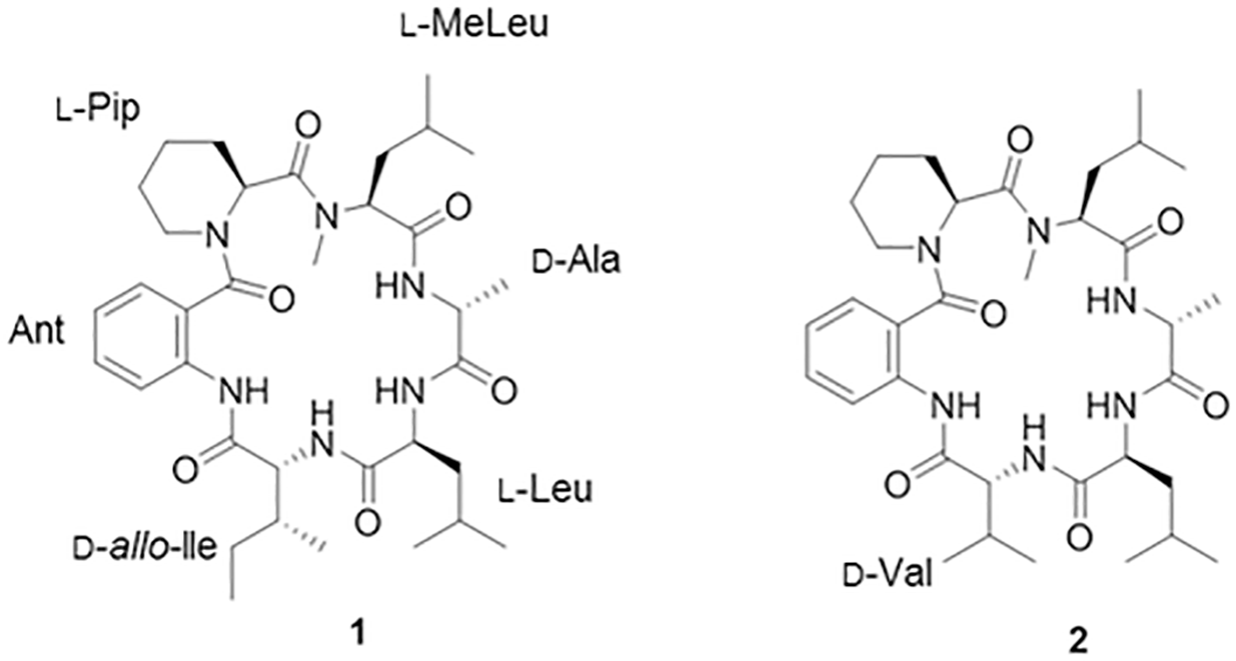
Chemical structures of PF1171A (1) and PF1171C (2).

The EtOAc extract of *Penicillium* sp. FN070315 was subjected to repeated silica gel, and RP-C_18_-gel column chromatography, followed by semipreparative RP-HPLC, to yield two cyclic peptides (**1** and **2**) (Fig 1). These two known compounds were identified as PF1171A (**1**) [18, 19] and PF1171C (**2**) [19, 20] by comparing their physicochemical and spectroscopic data ([α]_D,_^1^H and ^13^C NMR, HR-ESIMS) with the values reported in the literature (Supporting Information). The absolute configurations of the amino acids in **1** and **2** were determined by Marfey’s method after acid hydrolysis (table in S1 Table and table in S2 Table).

### The effects of PF1171A (1) and PF1171C (2) on the viability of human umbilical vein endothelial cells

**Fig 2:**
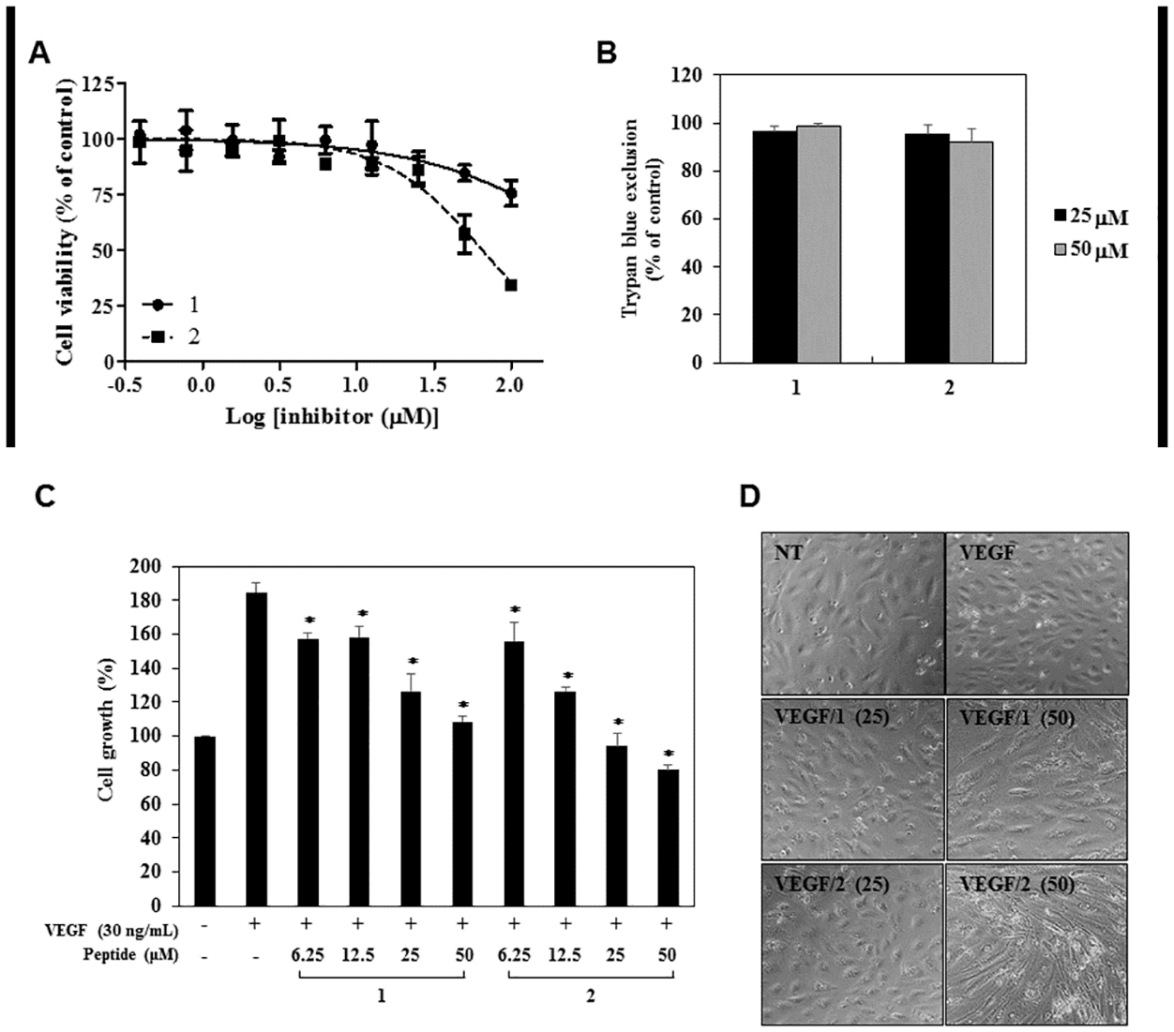
The antiproliferative activities of PF1171A (1) and PF1171C (2) on the HUVECs. (A, B) The effects of **1** and **2** on the viability of the HUVECs. (A) Cells were treated with various concentrations of **1** and **2** (0–100 μM) for 72 h, and cell viability was measured by the MTT colorimetric assay. (B) Cells were treated with **1** and **2** (25 and 50 μM) and incubated for 72 h. Cell viability was measured by the trypan blue assay. (A, B) Data were presented as percentage relative to DMSO-treated control (% of control). Each value represents the mean ± SE from three independent experiments. (C, D) The effects of **1** and **2** on the VEGF-induced growth of HUVECs. Serum starved HUVECs were stimulated by VEGF (30 ng/mL) with or without **1** and **2** (0–50 μM) for 72 h. (C) Cell growth was measured using the MTT colorimetric assay. *P < 0.01 versus the VEGF control. Each value represents the mean ± SE from three independent experiments. (D) The effects of **1** and **2** on the morphology of HUVECs.

In order to determine the optimum doses of **1** and **2** with no cytotoxicity for angiogenesis assays, various concentrations of **1** and **2** were applied to human umbilical vein endothelial cells (HUVECs) and viability assays were performed using the MTT colorimetric assay and trypan blue exclusion method. As shown in Fig 2A, **1** and **2** inhibited the viability of HUVECs with different sensitivities relative to DMSO-treated control. Compound **2** suppressed the viability of HUVECs with an IC_50_ value of 64.53 μM, whereas **1** showed a relatively low inhibitory rate of 24% against the viability of HUVECs even at a treatment concentration of 100 μM. However, the trypan blue assay revealed that treatment with up to 50 μM of **1** and **2**, compared with DMSO-treated control, did not have cytotoxic effects on the HUVECs (Fig 2B). Based on these results, the various *in vitro* angiogenesis assays were performed using the treatment concentrations of less than 50 μM of **1** and **2** at which no cytotoxicity was observed.

### The antiangiogenic activities of PF1171A (1) and PF1171C (2) on HUVECs

The key stages in the process of angiogenesis include the stimulation of endothelial cells (ECs) by angiogenic factors such as vascular endothelial growth factor (VEGF), degradation of the capillary basal lamina by activated ECs, migration and proliferation of ECs, vascular tube formation, and maturation. We therefore examined the effects of **1** and **2** on several angiogenic processes of HUVECs induced by VEGF.

We first investigated the effects of **1** and **2** on the proliferation of HUVECs stimulated by VEGF with the MTT colorimetric assay. As shown in Fig 2C, the peptides dose-dependently inhibited the VEGF-induced proliferation of HUVECs. Treatment with **1** and **2** also caused marked morphological changes (Fig 2D). Control HUVECs with VEGF treatment showed a cobblestone-like growth pattern, whereas the HUVEC morphology treated with VEGF and the peptides converted to a spindle fibroblast-like morphology.

**Fig 3:**
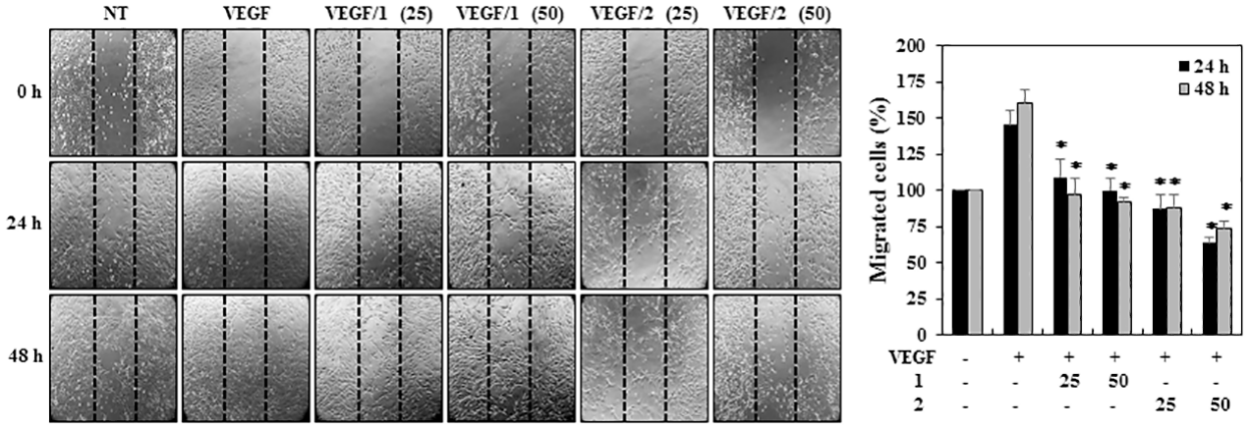
The effects of PF1171A (1) and PF1171C (2) on the migration of HUVECs using the wound healing assay. Confluent scrape-wounded HUVEC monolayers in serum-free media were incubated with **1** and **2** (25 and 50 μM) in the presence of VEGF (30 ng/mL). The perimeter of the area with a central cell-free gap was confirmed at the indicated time intervals (0, 24, and 48 h) and the cells migrated into the gap were counted under an optical microscope. Dotted black lines indicate the edge of the gap at 0 h. *P < 0.01 versus the VEGF control. Each value represents the mean ± SE from three independent experiments.

A wound healing assay was used to investigate the effects of **1** and **2** on the migration of the HUVECs. Briefly, confluent scrape-wounded HUVEC monolayers were incubated with VEGF in the presence or absence of **1** or **2**, and the number of cells that had migrated into the wound region was assessed 24 and 48 h later. As shown in Fig 3, treatment with **1** and **2** at 25 μM effectively inhibited the VEGF-induced migration of the HUVECs at both 24 and 48 h.

**Fig 4:**
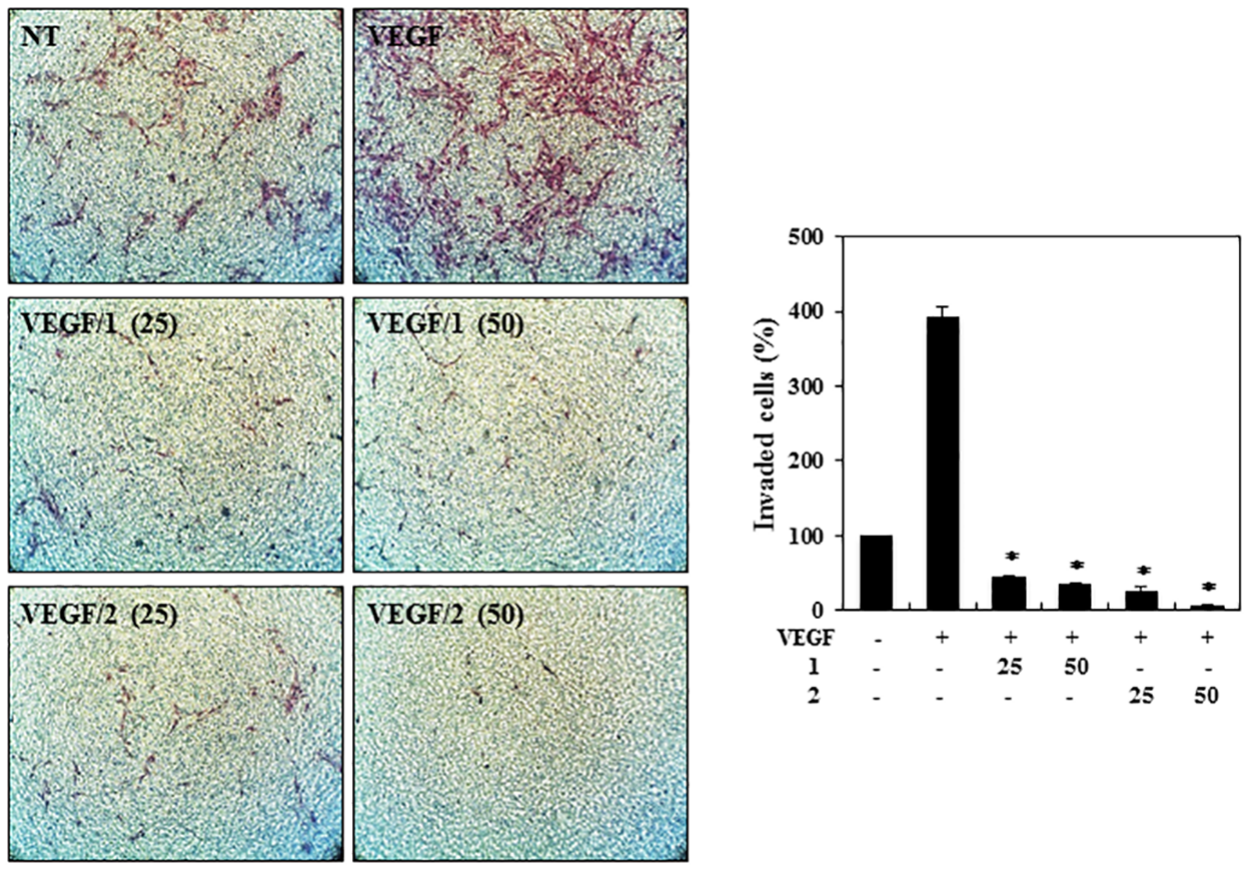
The effects of PF1171A (1) and PF1171C (2) on the invasion of the HUVECs. Serum-starved HUVECs were stimulated with VEGF (30 ng/mL) in the presence or absence of **1** and **2** (25 and 50 μM). The basal levels of invasiveness of the HUVECs that were incubated in serum-free media without VEGF were normalized to 100%. *P < 0.01 versus the VEGF control. Each value represents the mean ± SE from three independent experiments.

We next performed a chemoinvasion assay to evaluate the ability of VEGF-stimulated HUVECs to pass through Matrigel and a transwell membrane barrier a in the presence or absence of **1** or **2**. As shown in Fig 4, both peptides completely suppressed the VEGF-induced invasion of the HUVECs at 25 μM.

**Fig 5:**
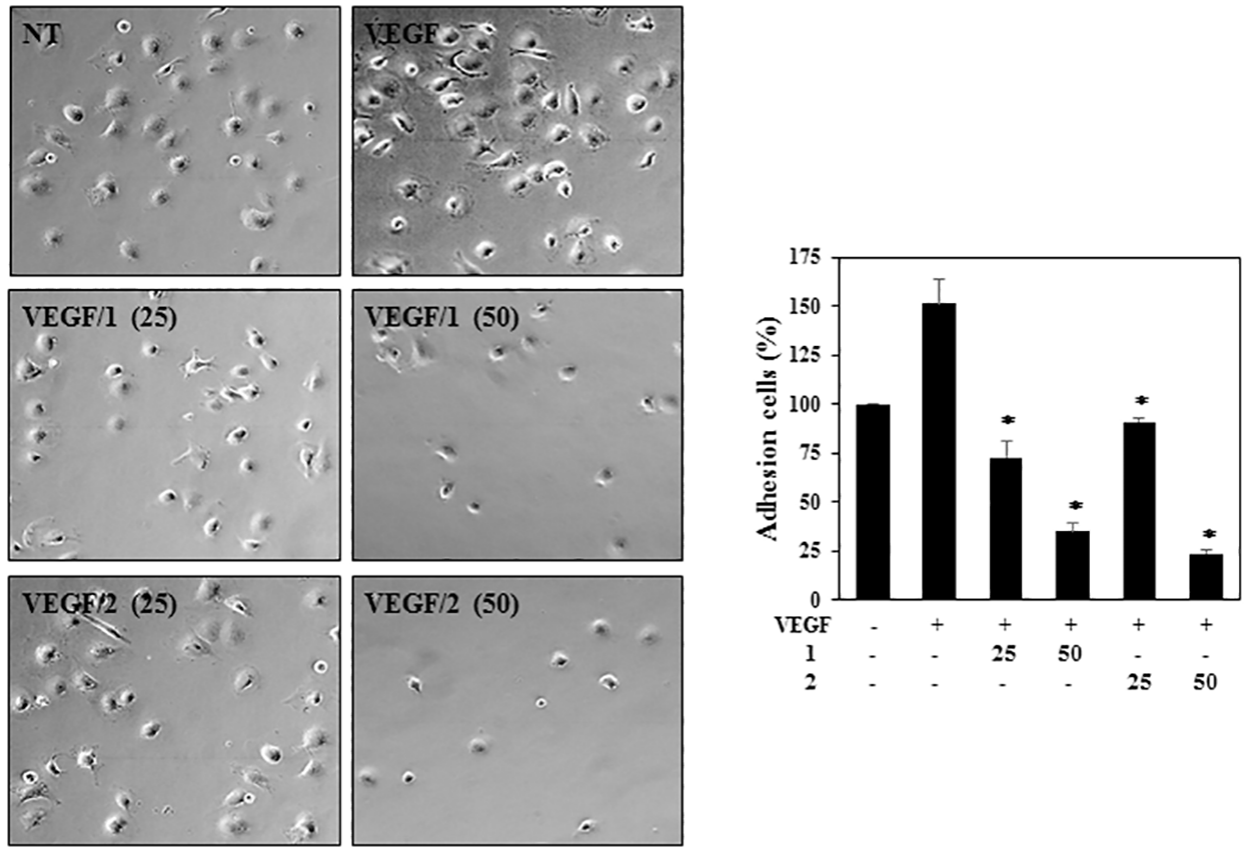
The effects of PF1171A (1) and PF1171C (2) on the adhesion of the HUVECs. Serum-starved HUVECs pretreated with VEGF (30 ng/mL) in the presence or absence of **1** and **2** (25 and 50 μM) were added to pre-coated wells to laminin and then incubated for 2 hrs. The basal levels of adhesion of the HUVECs that were incubated in serum-free media without VEGF were normalized to 100%. *P < 0.01 versus the VEGF control. Each value represents the mean ± SE from three independent experiments.

In order to determine the effects of **1** and **2** on endothelial cell adhesion, the adhesion of HUVECs to extracellular matrix (ECM) protein was assessed. The results showed that the cell adhesion of VEGF-induced HUVECs to laminin was inhibited by treatment with **1** and **2** in a dose-dependent manner (Fig 5).

**Fig 6:**
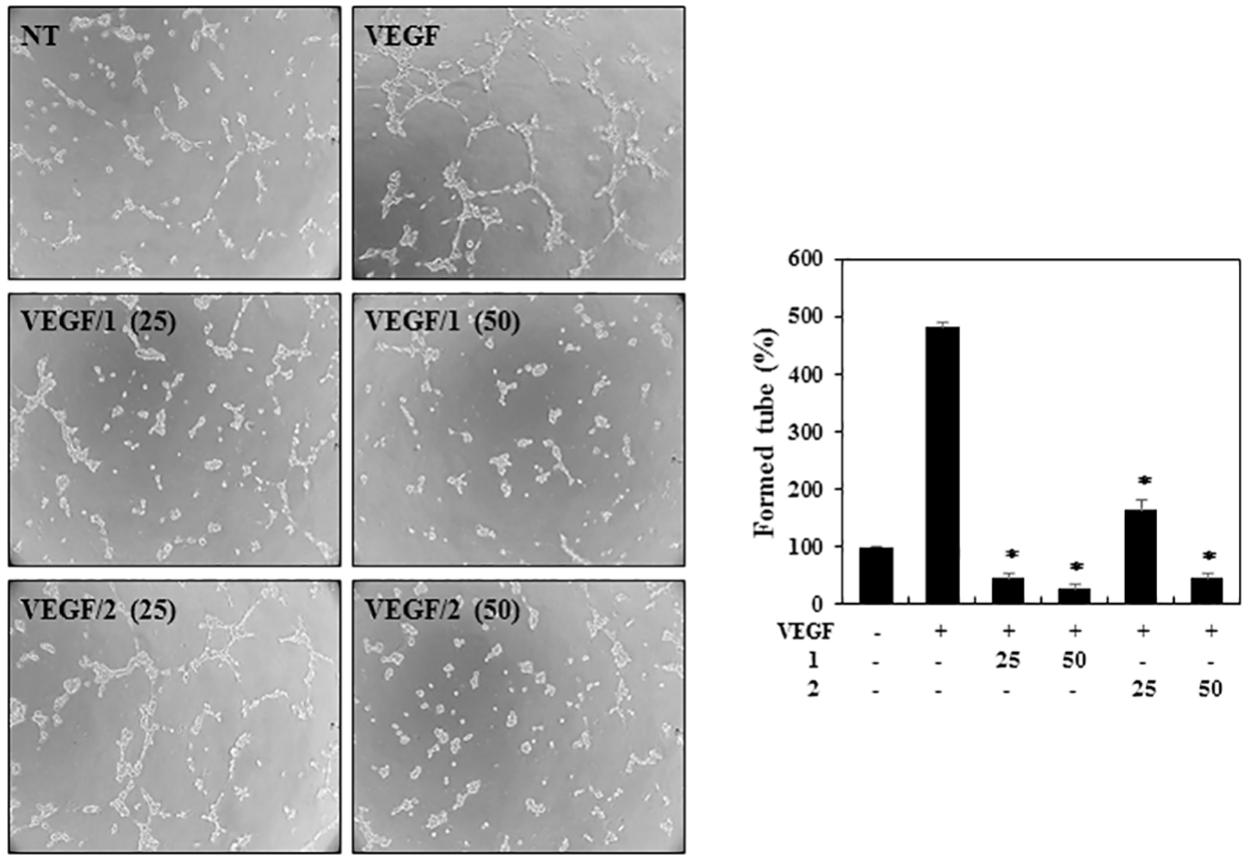
The effects of PF1171A (1) and PF1171C (2) on the tube-forming ability of the HUVECs. Serum-starved HUVECs were stimulated with VEGF (30 ng/mL) in the presence or absence of **1** and **2** (25 and 50 μM). The basal levels of capillary tube formation of the HUVECs that were incubated in serum-free media without VEGF were normalized to 100%. *P < 0.01 versus the VEGF control. Each value represents the mean ± SE from three independent experiments.

For a further investigation of the antiangiogenic effects, the tube formation ability of the HUVECs was assessed. HUVECs were plated on the surface of Matrigel, and VEGF stimulated the formation of capillary-like structures by HUVECs. However, treatment with the indicated concentrations of **1** and **2** significantly decreased the VEGF-induced tube formation of the HUVECs (Fig 6). Taken together, these results show that **1** and **2** potently inhibit VEGF-induced angiogenesis of HUVECs at subtoxic doses.

### The *in vivo* antiangiogenic activities of PF1171A (1) and PF1171C (2)

**Fig 7:**
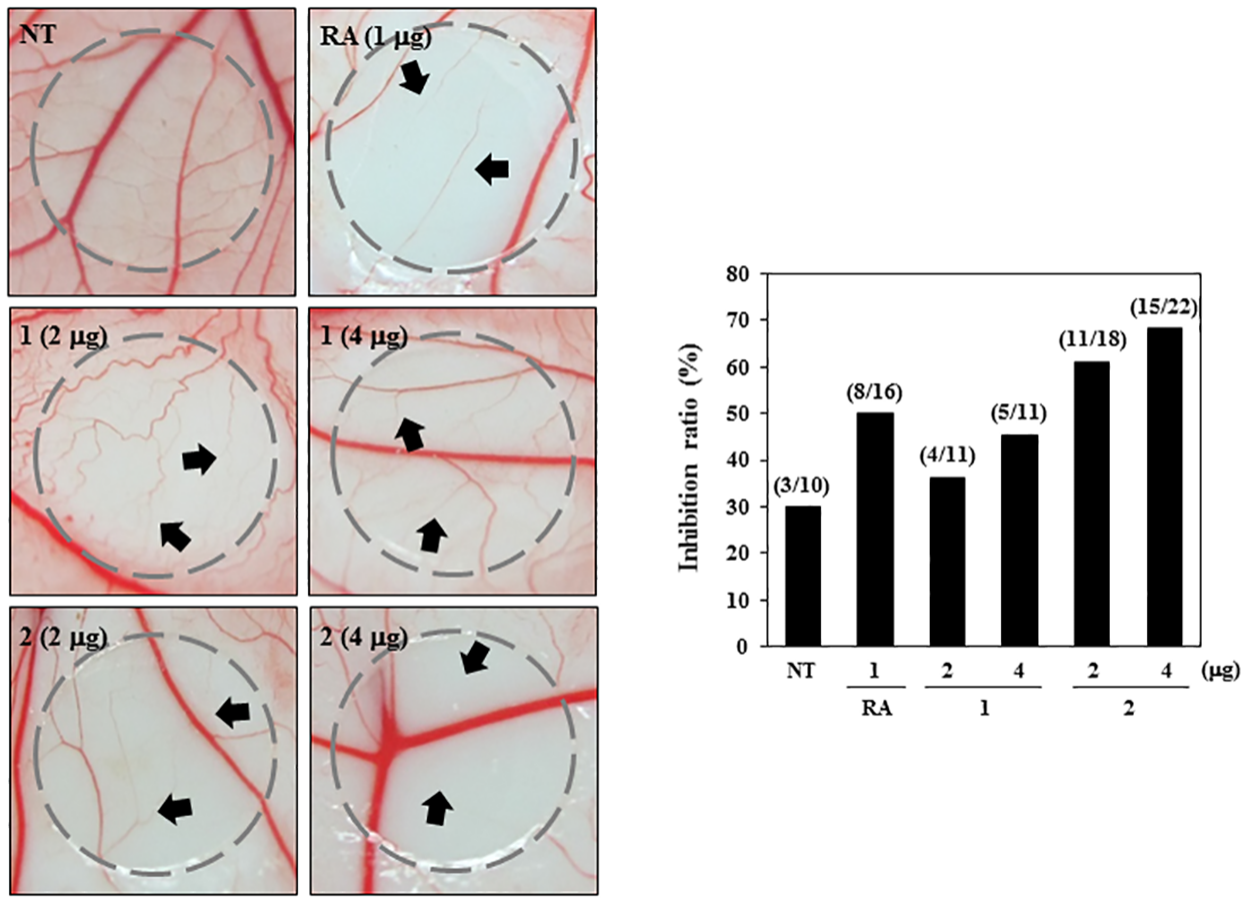
The effects of PF1171A (1) and PF1171C (2) on angiogenesis in CAMs *in vivo*. Fertilized chick eggs were maintained in a humidified incubator at 37°C. At embryonic day 4.5, coverslips loaded with vehicle alone, RA (1 μg) or **1** or **2** (2 and 4 μg) were applied to the CAM surface. Two days later, the chorioallantois was visualized under a microscope. The presence of an avascular zone (arrows) in the treated CAMs was scored as a positive response. Calculation (inhibition ratio) was based on the proportion of positive eggs relative to the total number of live eggs.

To validate the antiangiogenic effects of **1** and **2** *in vivo*, we did a chorioallantoic membrane (CAM) assay. Retinoic acid (RA) is a known antiangiogenic compound. In this assay, vehicle alone and RA were used as negative and positive controls for antiangiogenic responses, respectively. Coverslips containing vehicle alone, RA, **1** or **2** were placed on the CAM surface, and angiogenesis zones were observed under a microscope. Retinoic acid (RA) was used as a positive control for the antiangiogenic responses. As shown in Fig 7, the inhibition of angiogenesis by RA was 50% (n = 16), and the inhibition of the negative control coverslips was 30% (n = 10). On the other hand, **2** considerably inhibited the angiogenesis of CAM (61% at 2 μg/egg, n = 18; 68% at 4 μg/egg, n = 22) compared with the control without toxicity against pre-existing vessels. Whereas **1** exhibited mild antiangiogenic activity (36% at 2 μg/egg, n = 11; 45% at 4 μg/egg, n = 11). These data reveal that **1** and **2** possess promising antiangiogenic potential.

### The inhibitory effects of PF1171A (1) and PF1171C (2) on the tumor cell-induced angiogenesis

**Fig 8:**
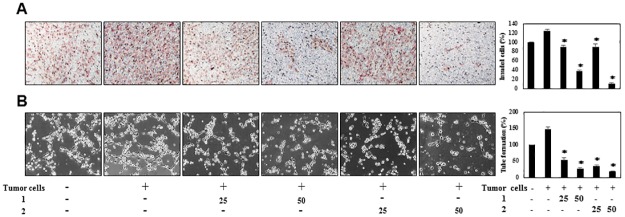
The effects of PF1171A (1) and PF1171C (2) on the tumor cell-induced angiogenesis. (A, B) HepG2 cells were treated with **1** and **2** (25 and 50 μM) for 24 h, and then the conditioned media (CM) were concentrated by Amicon ultra centrifugal filters. The CM were used in the *in vitro* endothelial cell invasion and tube formation assays. The basal levels of the invasion and tube formation of HUVECs that treated the CM without HepG2 cells were normalized to 100%. *P < 0.01 versus the CM from untreated HepG2 cells. Each value represents the mean ± SE from three independent experiments.

Next, we investigated the effects of **1** and **2** on the angiogenesis-promoting potential of HepG2 hepatoma cells. The conditioned media (CM) from the HepG2 cells incubated in the presence or absence of **1** and **2** were collected, and their effects were investigated using the *in vitro* angiogenesis assays. The CM from the tumor cells was found to activate the invasion of HUVECs, whereas those from HepG2 cells treated with **1** and **2** blocked the tumor cell-induced invasion of HUVECs in a dose-dependent manner (Fig 8A). To further verify their activities to tumor cell-induced angiogenesis, we also assessed the effects of **1** and **2** on the tube formation of HUVECs induced by HepG2 cells. As shown in Fig 8B, the CM from HepG2 cells significantly induced the tube formation of HUVECs compared to control (medium only). However, the peptides-treated CM from HepG2 cells blocked the stimulated tube formation of HUVECs in a dose-dependent manner, implying that **1** and **2** might inhibit tumor cell-induced angiogenesis.

### Downregulation of VEGFR2 and HIF-1α by PF1171A (1) and PF1171C (2)

**Fig 9:**
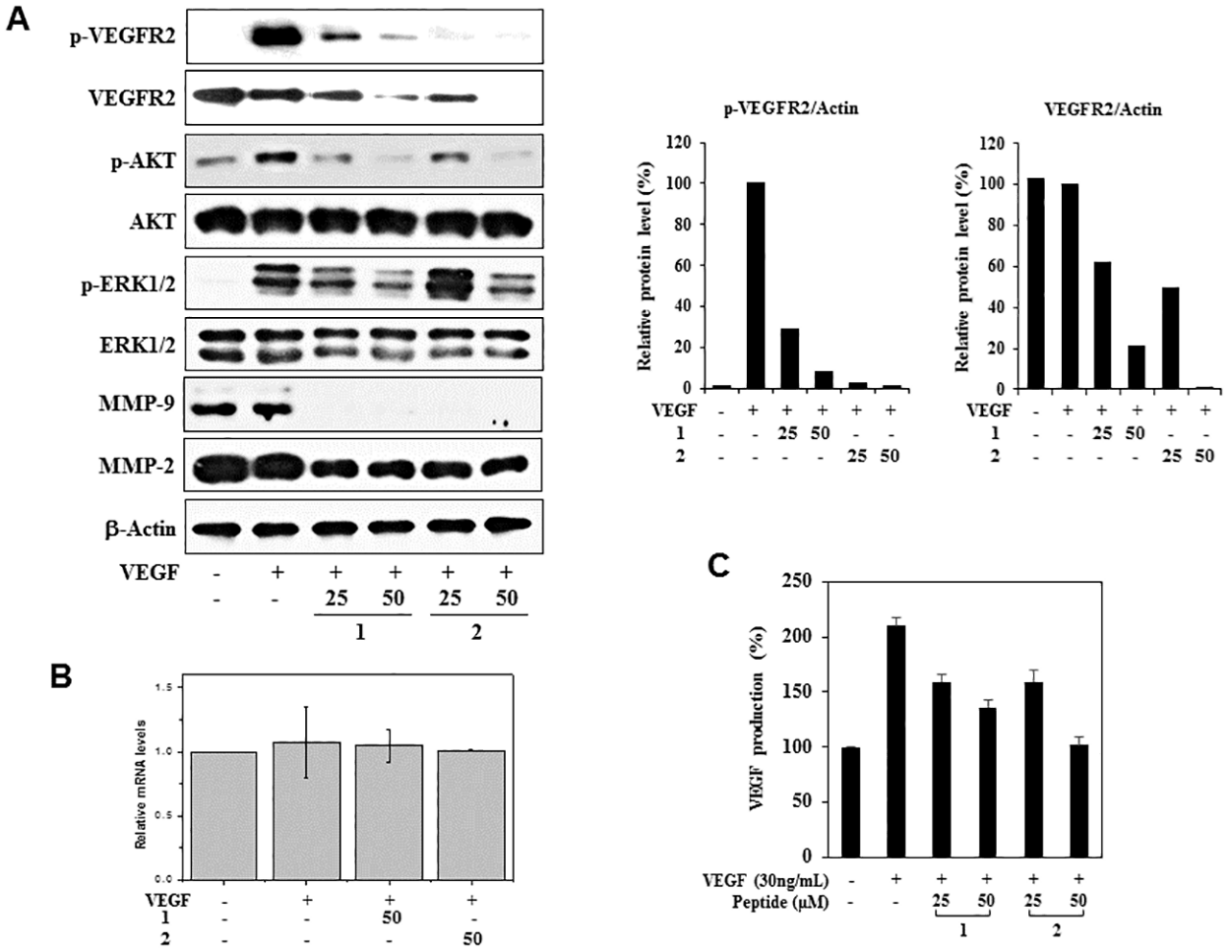
The effects of PF1171A (1) and PF1171C (2) on the VEGFR2 signaling and MMPs expression. (A, B) Serum-starved HUVECs were pretreated with **1** and **2** (25 and 50 μM) for 6 h and then stimulated with VEGF (30 ng/mL) for 5 min. (A) Downregulation of VEGFR2 signaling and MMPs by **1** and **2**. Protein levels were detected by Western blot analysis using specific antibodies and further quantified by densitometry. The levels of β-actin were used as an internal control. (B) VEGFR2 mRNA levels were determined by real-time quantitative PCR. Values are expressed as mean ± SD from four separated experiments. (C) The effects of **1** and **2** on the VEGF secretion levels of HUVECs stimulated by VEGF for 72 h.

To elucidate the possible mechanisms of angiogenesis inhibition by **1** and **2**, we investigated their effects on the phosphorylation of VEGF receptor 2 (VEGFR2) in the HUVECs. As shown in Fig 9A, **1** and **2** remarkably suppressed the phosphorylation of VEGFR2 induced by VEGF. In addition, they also reduced the total protein levels of VEGFR2, suggesting that the inhibitory effects on VEGFR2 expression may affect the VEGF-induced VEGFR2 phosphorylation. However, **1** and **2** had no effect on VEGFR2 mRNA expression (Fig 9B). Therefore, **1** and **2** may inhibit VEGFR2 expression at the level of translation, but not transcription. **1** and **2** also decreased the VEGF-induced AKT and ERK1/2 phosphorylation without affecting the total protein levels, indicating that **1** and **2** exhibit the antiangiogenic activities by inhibiting VEGFR2-mediated downstream signaling cascades (Fig 9A). Moreover, they suppressed the protein expression of matrix metalloproteases (MMPs), including MMP-2 and MMP-9, which degrade ECM to allow for endothelial sprouting (Fig 9A). We further analyzed the VEGF secretion levels by enzyme-linked immunosorbent assay (ELISA). Treatment with **1** and **2** significantly inhibited the VEGF production of HUVECs stimulated by VEGF (Fig 9C). These results suggest that **1** and **2** could suppress the endogenous expression of VEGF, followed by the activation of VEGFR2.

**Fig 10:**
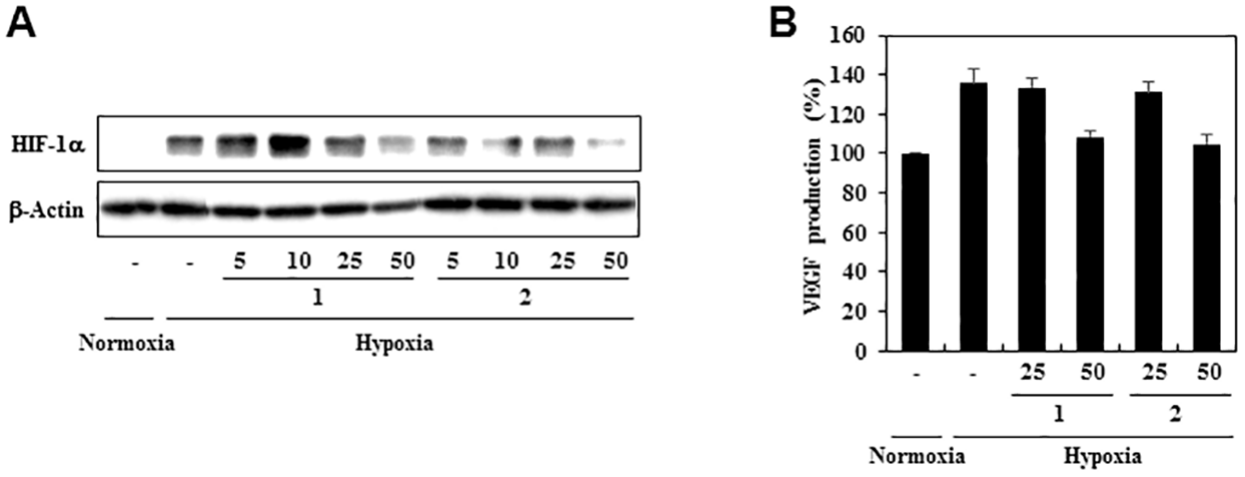
The effects of PF1171A (1) and PF1171C (2) on the HIF-1α activity. (A) HIF-1α inhibitory activities of **1** and **2**. HepG2 cells were pretreated with **1** and **2** for 30 min at the indicated concentrations and then exposed to 1% O_2_ for 8 h. Protein levels were detected by Western blot analysis and the levels of β-actin were used as an internal control. (B) The effects of **1** and **2** on VEGF expression. The concentration of VEGF protein in the culture supernatant obtained from (A) was determined by a VEGF specific ELISA.

**Fig 11:**
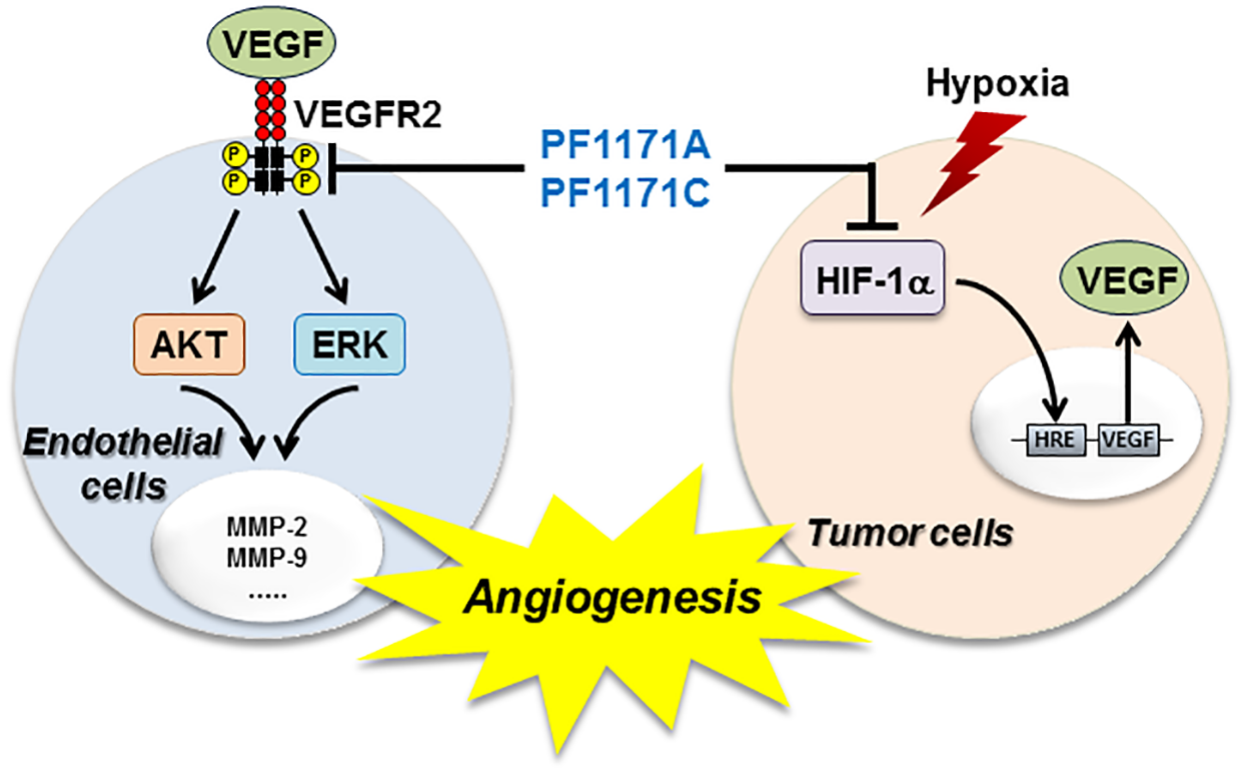
Schematic diagram of the mechanism by which PF1171A (1) and PF1171C (2) inhibit angiogenesis. The cyclic hexapeptides could suppress angiogenesis by modulating at least two angiogenic pathways, namely the blockade of VEGFR2 signal transduction and inhibition of the expression of HIF-1α/VEGF.

Hypoxia inducible factor-1α (HIF-1α) activation in hypoxic tumor cells is an important stimulus for tumor angiogenesis through the regulation of the expression of proangiogenic genes such as VEGF. To assess the effects of **1** and **2** on the expression of the HIF-1α under hypoxic condition, the levels of HIF-1α protein were measured in the human hepatoma cell line the HepG2, a hypervascularized tumor. As shown in Fig 10A, exposure of these cells to 50 μM of **1** and **2** significantly reduced the levels of HIF-1α protein under hypoxic condition. We further determined the effects of **1** and **2** on the expression of VEGF induced by hypoxia. Treatment with **1** and **2** at 50 μM decreased VEGF production in HepG2 cells under hypoxic condition (Fig 10B). Thus, these results suggest that **1** and **2** might efficiently block angiogenesis by downregulating both VEGFR2 and HIF-1α activities (Fig 11).

In the past few decades, many angiogenesis inhibitors have been created to reduce many tumors’ progression and invasiveness. Avastin (bevacizumab), in combination with 5-fluorouracil-based chemotherapy regimens, is now used to treat patients with metastatic colorectal cancer in the United States and Europe. However, it is of great interest to identify new angiogenesis inhibitors, as several small molecules with antiangiogenic activity are not clinically useful for toxicity [21,22]. Therefore, continued research to discover novel antiangiogenic scaffolds is urgently needed to overcome the limitations of the current angiogenesis inhibitors. Some natural products, including multiple members of the macrolide latrunculins, the macrocyclic oxaquinolizidine alkaloid araguspongine C, and the sesquiterpene quinone puupehenone, showed promising results in primary and secondary angiogenesis screening modules [23]. Previously other groups have reported the anticancer and antiangiogenic activities of several peptides from natural products [24–26]. These results are consistent with those of PF1171A (**1**) and C (**2**) in that similar changes in cell morphology, decreases in VEGFR2 expression and the other signaling changes in HUVECs reported before were also observed here. Further, PF1171A (**1**) and C (**2**) can share a common mechanism of action, and both peptides are composed of hydrophobic amino acids that will allow for permeability through the cell membrane. These hits inhibited vascular endothelial growth factor (VEGF)-mediated endothelial tube-like formation, with minimal cytotoxicity at relevant doses. In this study, we found that PF1171A (**1**) and PF1171C (**2**) exhibits antiangiogenic activity both in vitro and in vivo with no cytotoxicity. Furthermore, our results showed that PF1171A(**1**) and PF1171C (**2**) may inhibit tumor angiogenesis by at least two mechanisms: the blockade of VEGFR2 signaling in endothelial cells and suppression of the expression of the key transcription factor HIF-1α in tumor cells.

## Conclusions

We isolated two cyclic hexapeptide, PF1171A (**1**) and PF1171C (**2**) from *Penicillium* sp. FN070315 as the promising antiangiogenic bioactive molecules. The PF1171 family was originally isolated from the fermentation extract of the fungus *Hamigera avelanea* and suppressed apolipoprotein B secretion in HepG2 cells [20]. The compounds were members of cyclic peptides containing the nonproteinogenic anthranilic acid (Ant) and pipecolinic acid (Pip). Previously, PF1171A (**1**) and PF1171C (**2**) were evaluated for their paralytic activity against the larvae of the silkworm *Bombyx mori* [18]. To our knowledge, the antiangiogenic activity of PF1171A (**1**) and PF1171C (**2**) is reported for the first time in this study. We also found that PF1171A (**1**) and PF1171C (**2**) downregulated the expression of both VEGFR2 in endothelial cells and HIF-1α in tumor cells, thereby resulting in the suppression of VEGFR2 phosphorylation and VEGF production. These data suggest that PF1171A (**1**) and PF1171C (**2**) may inhibit tumor angiogenesis by modulating the upstream cellular mediators of VEGFR2 and HIF-1α pathways. Further investigation of the action mechanism and optimization of PF1171A (**1**) and PF1171C (**2**) might lead to finding new angiogenesis inhibitors.

## Supporting information

S1 Table LC/MS analysis of the FDLA derivatives of compound 1. (TIF)

S2 Table LC/MS analysis of the FDLA derivatives of compound 2. (TIF)

S1 Fig ^1^H NMR spectrum (500 MHz) of compound 1 in CDCl_3_. (TIF)

S2 Fig ^13^C NMR spectrum (125 MHz) of compound 1 in CDCl_3_. (TIF)

S3 Fig HRESIMS spectrum of compound 1. (TIF)

S4 Fig ^1^H NMR spectrum (500 MHz) of compound 2 in CDCl_3_. (TIF)

S5 Fig ^13^C NMR spectrum (125 MHz) of compound 2 in CDCl_3_. (TIF)

S6 Fig HRESIMS spectrum of compound 2. (TIF)

S1 File Physico-chemical properties of compounds 1 and 2. (PDF)
