# Supplementary figures and images for: Two cyclic hexapeptides from Penicillium sp. FN070315 with antiangiogenic activities
Source: PLoS One. 2017 Sep 26;12(9):e0184339. doi: 10.1371/journal.pone.0184339 (PMC5614539; doi:10.1371/journal.pone.0184339)

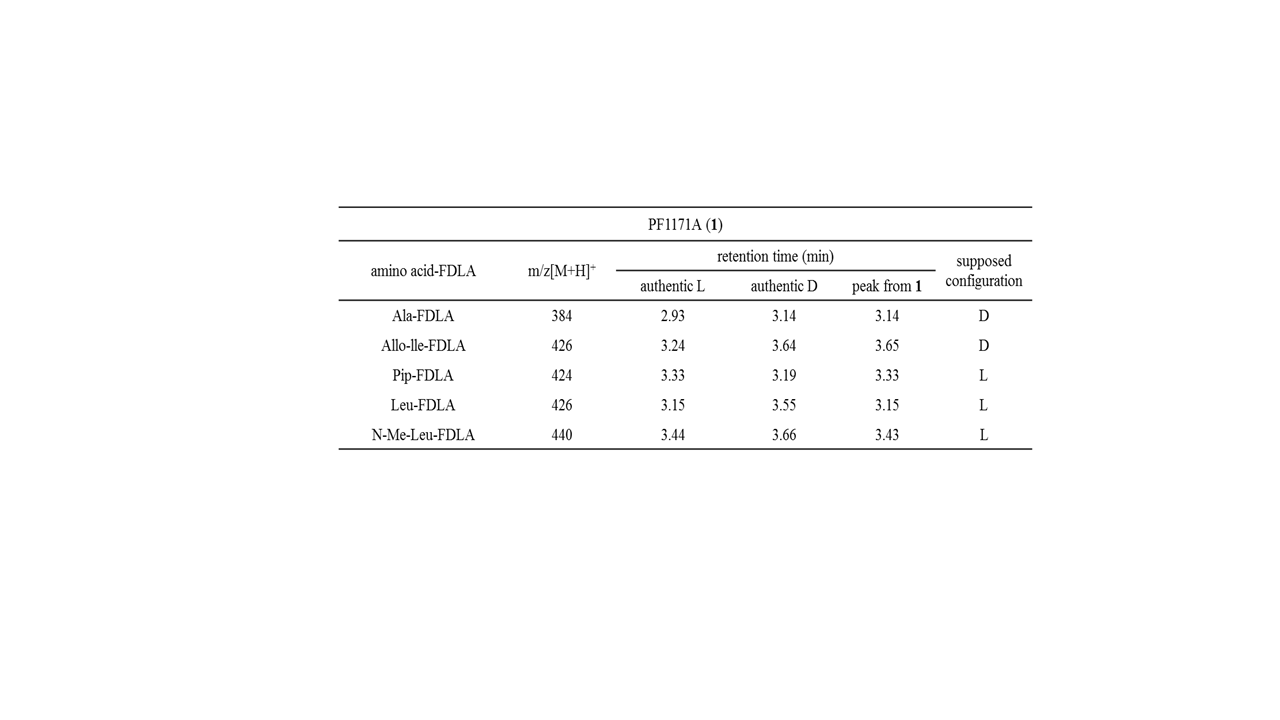

Supplement: S1 Table — (TIF) [file pone.0184339.s001.tif]

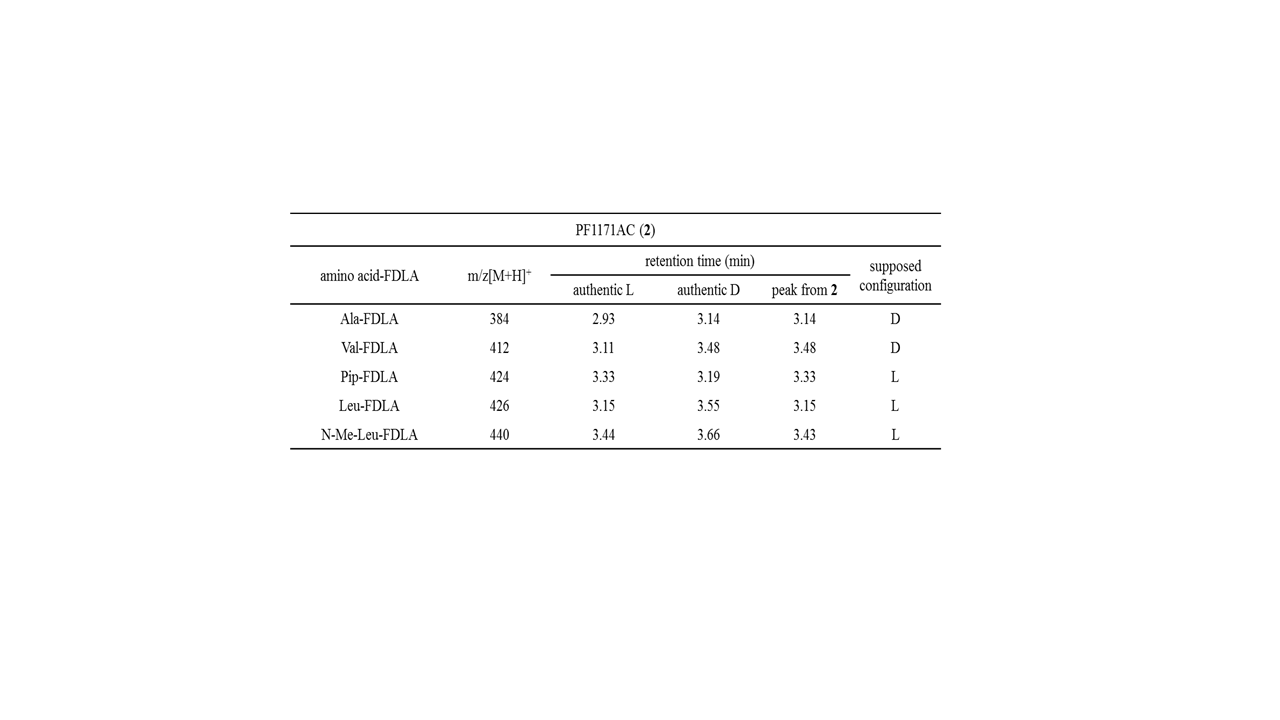

Supplement: S2 Table — (TIF) [file pone.0184339.s002.tif]

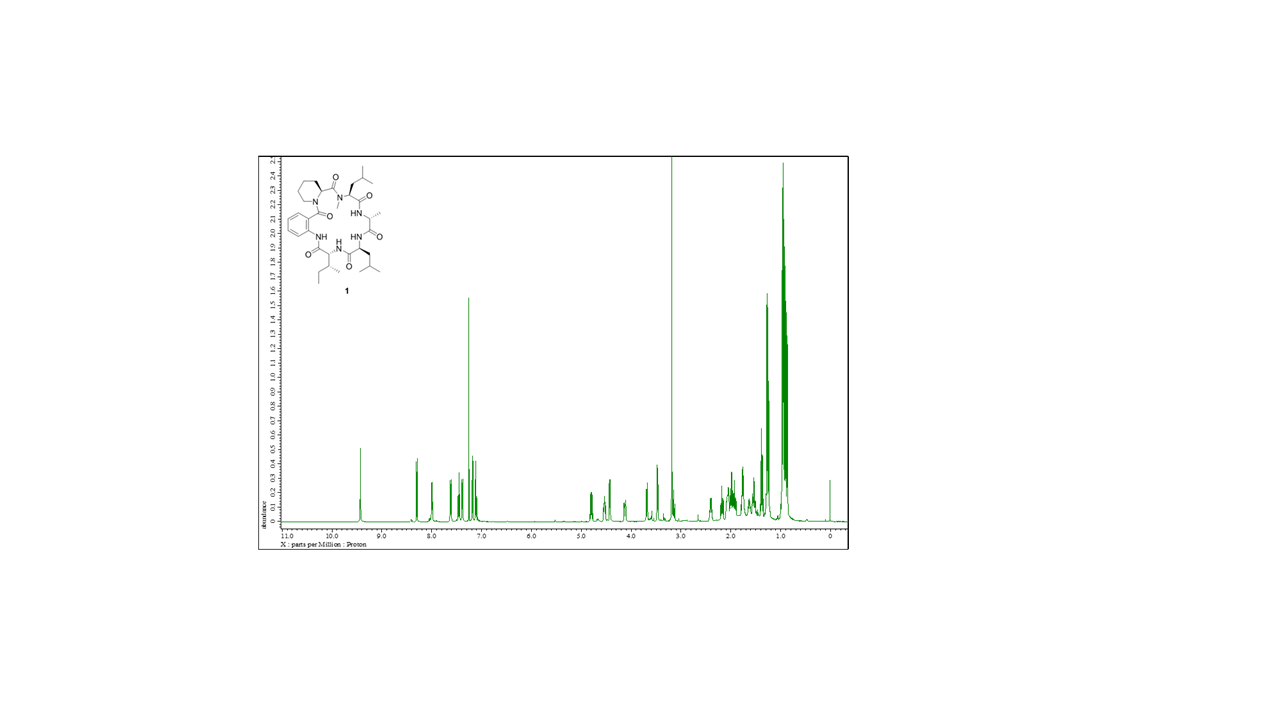

Supplement: S1 Fig — (TIF) [file pone.0184339.s003.tif]

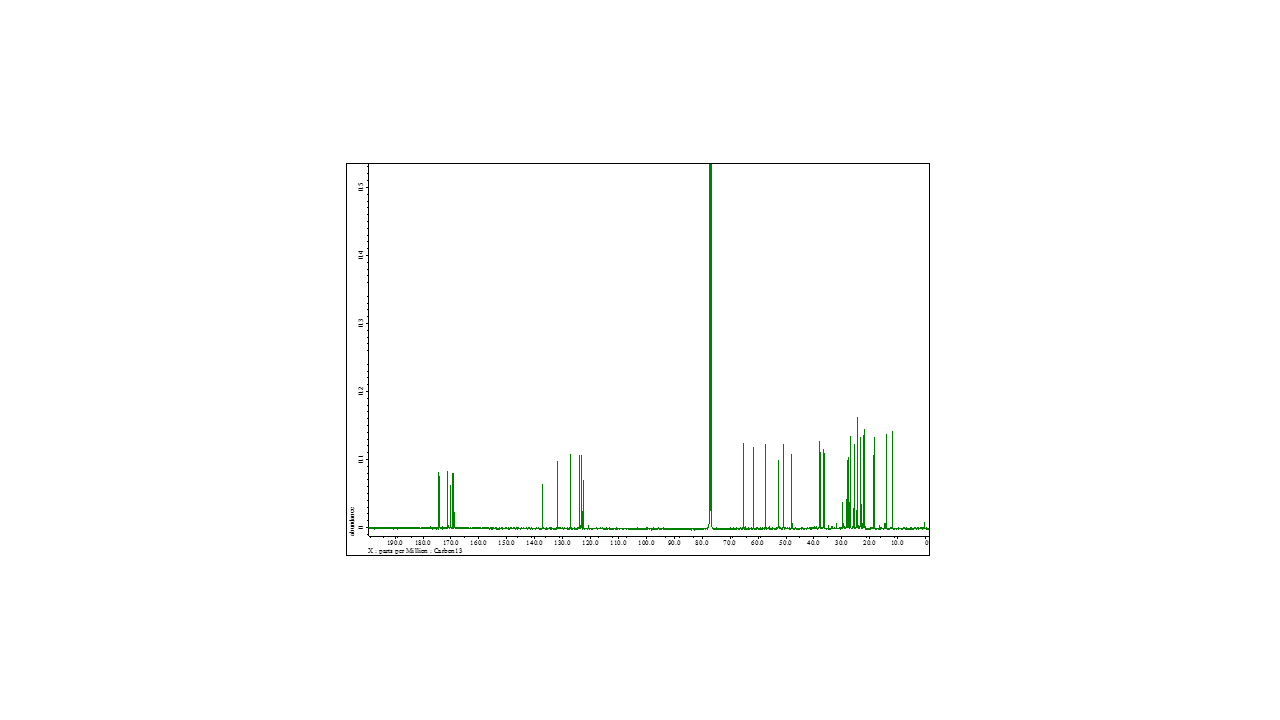

Supplement: S2 Fig — (TIF) [file pone.0184339.s004.tif]

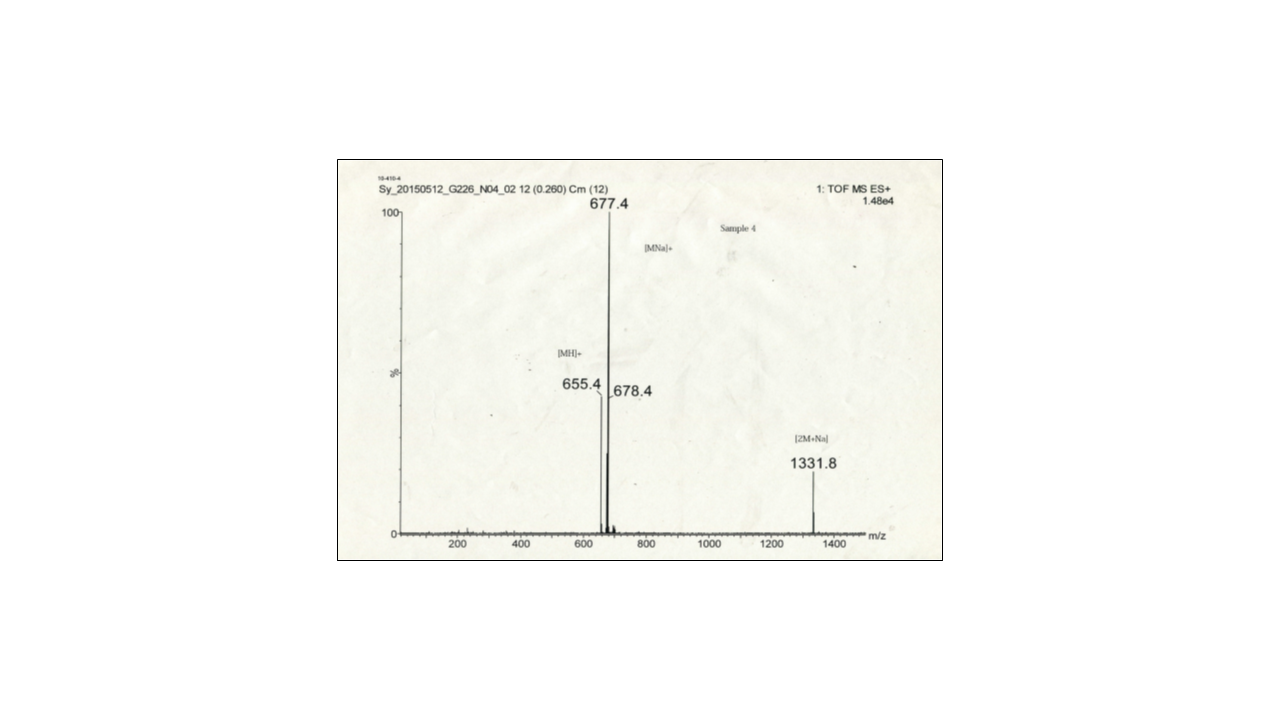

Supplement: S3 Fig — (TIF) [file pone.0184339.s005.tif]

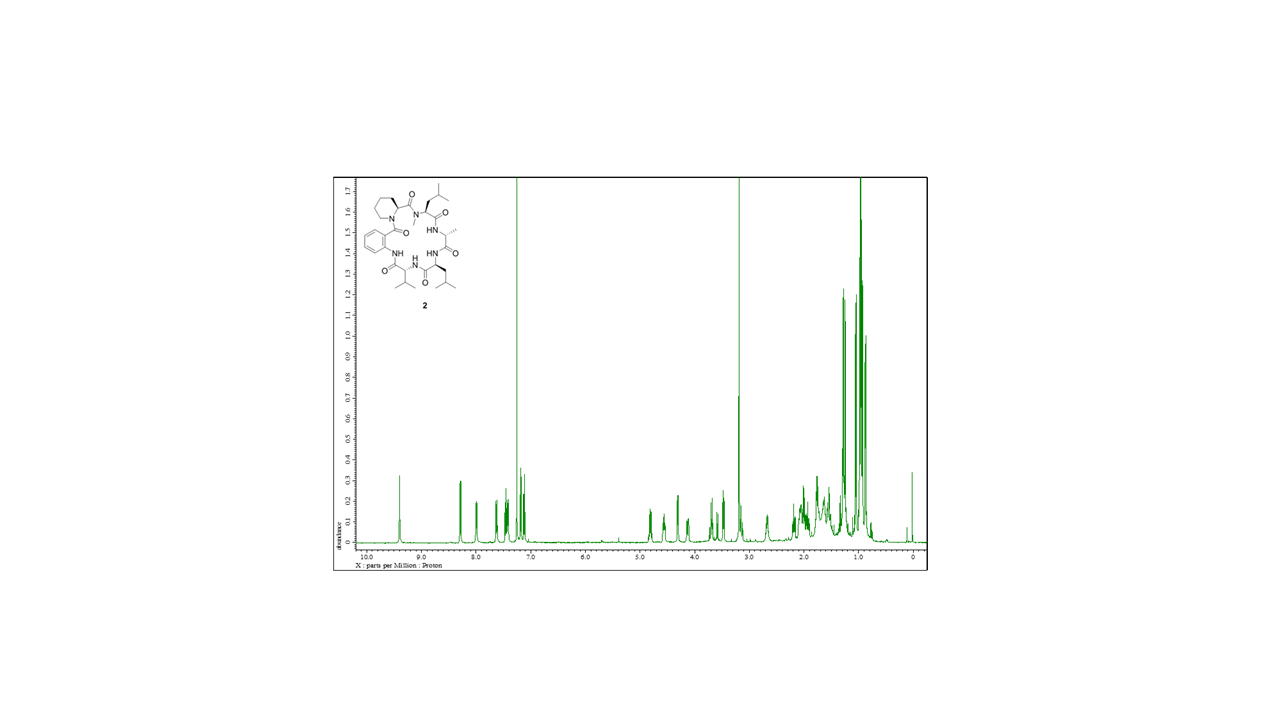

Supplement: S4 Fig — (TIF) [file pone.0184339.s006.tif]

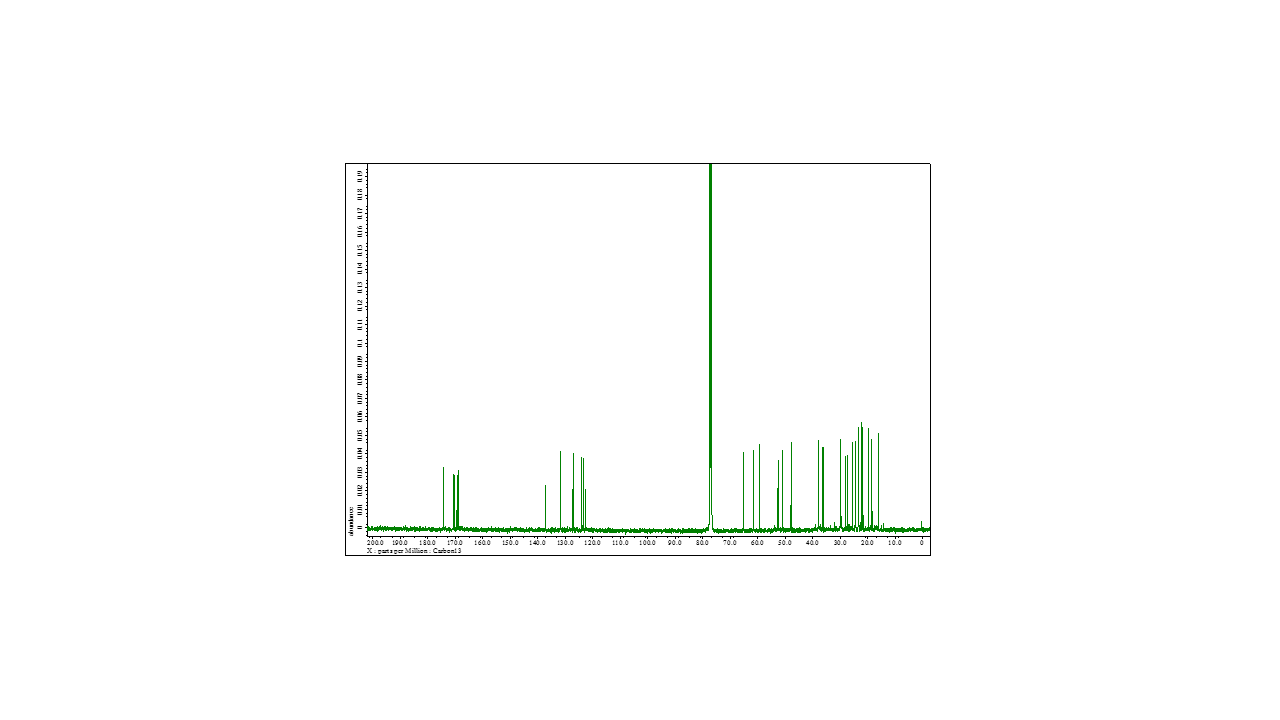

Supplement: S5 Fig — (TIF) [file pone.0184339.s007.tif]

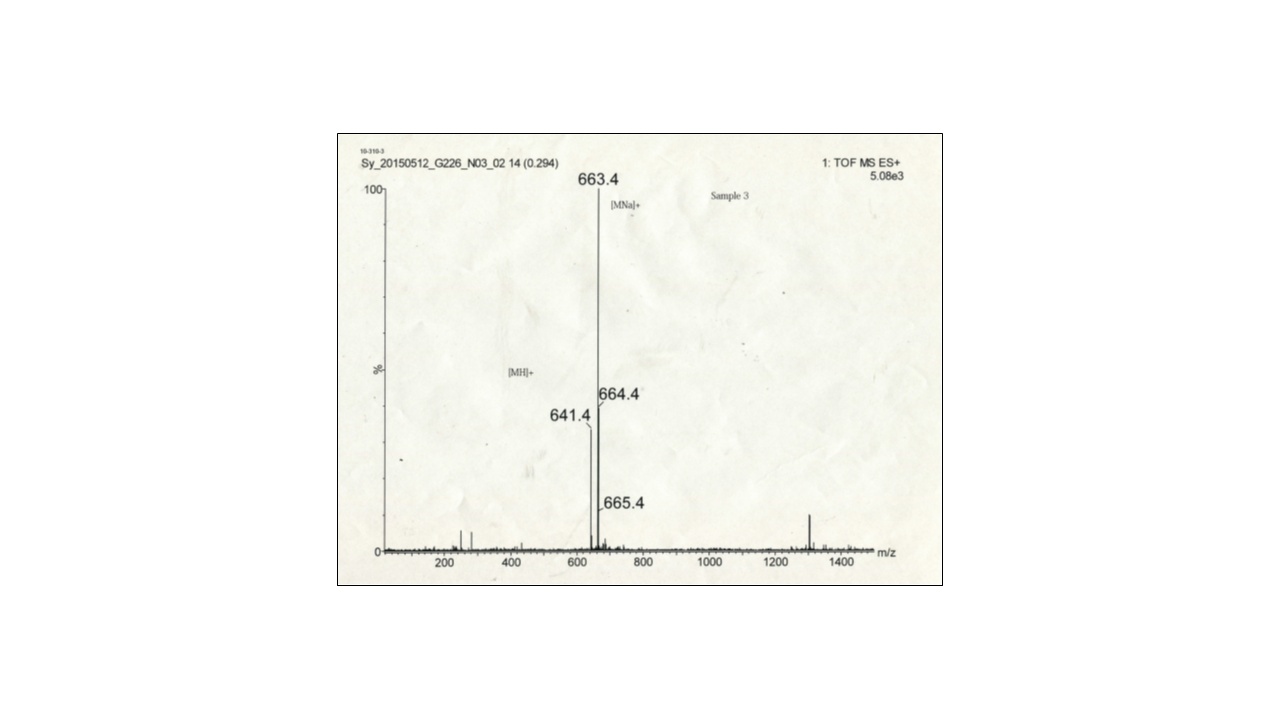

Supplement: S6 Fig — (TIF) [file pone.0184339.s008.tif]
