# Supplementary material for: Two cyclic hexapeptides from Penicillium sp. FN070315 with antiangiogenic activities
Source: PLoS One. 2017 Sep 26;12(9):e0184339. doi: 10.1371/journal.pone.0184339 (PMC5614539; doi:10.1371/journal.pone.0184339)
Supplement: S1 File — (PDF) [file pone.0184339.s009.pdf]

Physico-chemical properties of compound 1 and 2

PF1171A (**1**): white solid; $[\alpha]_{589}^{25}$ +31° (c 0.15, MeOH); ^{1}H-NMR (500 MHz, $CDCl_3$) δ: 0.87 (3H, d, J = 6.0 Hz, Leu-δ), 0.90 (3H, d, J = 7.2 Hz, *allo*-Ile-γ), 0.91 (3H, t, J = 7.2 Hz, *allo*-Ile-δ), 0.94 (3H, d, J = 5.2 Hz, Leu-δ'), 0.95, (3H, d, J = 7.2 Hz, MeLeu-δ'), 0.97 (3H, d, J = 6.8 Hz, MeLeu-δ), 1.27 (3H, d, J = 6.8 Hz, Ala-β), 1.38 (2H, m, *allo*-Ile-γ'), 1.53 (1H, m, Leu-γ), 1.57 (2H, m, Pip-γ), 1.63 (1H, m, MeLeu-γ), 1.76 (2H, m, Leu-β, Pip-β), 1.91 (1H, m, MeLeu-β), 1.98 (1H, m, Leu-β), 2.04 (3H, m, Pip-β, Pip-δ), 2.18 (1H, m, MeLeu-β), 2.41 (1H, m, *allo*-Ile-β), 3.14 (1H, d, J = 3.0 Hz, Pip-ε), 3.17 (3H, s, MeLeu-*N*-Me), 3.47 (1H, m, MeLeu-α), 3.67 (1H, dd, J = 3.0, 11.5 Hz, Pip- α), 4.12 (1H, d, J = 13.5 Hz, Pip-ε), 4.43 (1H, dd, J = 3.0, 7.5 Hz, *allo*-Ile- α), 4.53 (1H, m, Leu- α), 4.80 (1H, m, Ala- α), 7.11 (1H, t, J = 7.5 Hz ABA-3), 7.18 (1H, d, J = 7.5 Hz, ABA-5), 7.39 (1H, d, J = 7.5 Hz, ABA-2), 7.46 (1H, t, J = 13.5 Hz, ABA-4), 7.62 (1H, d, J = 9.5 Hz, Ala-NH), 7.99 (1H, d, J = 8.0 Hz, Leu-NH), 8.30 (1H, d, J = 8.5 Hz, *allo*-Ile-NH), 9.43(1H, s, ABA-NH). ^{13}C-NMR (125 MHz, $CDCl_3$) δ: 11.9 (*allo*-Ile-δ), 14.0 (*allo*-Ile-γ), 18.5 (Ala-β), 21.8 (Leu-δ), 22.1 (MeLeu-δ), 23.2 (Leu-δ'), 23.4 (MeLeu-δ'), 24.5 (Leu-γ, Pip-β), 25.6 (MeLeu-γ), 26.9 (*allo*-Ile-γ'), 27.4 (Pip-γ), 28.1 (Pip-δ), 36.3 (Leu-β), 36.5 (*allo*-Ile- α), 37.8 (MeLeu-β), 37.9 (MeLeu-*N*-Me), 47.9 (Ala- α), 50.9 (Leu- α), 52.7 (Pip-ε), 57.3 (*allo*-Ile- α), 61.6 (Pip-α), 65.2 (MeLeu-α), 122.7 (ABA-1), 123.3 (ABA-3), 123.9 (ABA-2), 127.2 (ABA-5), 131.8 (ABA-4), 137.2 (ABA-6), 168.9 (Pip-CO), 169.3 (MeLeu-CO), 170.3 (ABA-CO), 171.1 (*allo*-Ile-CO), 174.1 (Leu-CO), 174.4 (Ala-CO).

PF1171C (**2**): white solid; $[\alpha]_{589}^{25}$ +26° (c 0.15, MeOH); ^{1}H-NMR (500 MHz, $CDCl_3$) δ: 0.87 (3H, d, J = 6.0 Hz, Leu-δ), 0.93 (3H, d, J = 7.2 Hz, Val-γ), 0.96 (6H, d, J = 6.8 Hz, I6.8 Hz, MeLeu-δ', Leu-δ), 0.97 (3H, d, J = 6.8 Hz, MeLeu-δ), 1.05, (3H, d, J = 7.2 Hz, Val-γ'), 1.28 (3H, d, J = 6.8 Hz, Ala-β), 1.54 (3H, m, Leu-γ, Pip-δ), 1.63 (1H, m, MeLeu-γ), 1.74 (1H, m, Pip-β), 1.76 (1H, m, Leu-β), 1.91 (1H, m, MeLeu-β), 2.00 (1H, m, Leu-β), 2.04 (1H, m, Pip-β), 2.08 (2H, m, Pip-γ), 2.17 (1H, m, MeLeu-β), 2.67 (1H, m, Val-β), 3.14 (1H, d, J = 3.0 Hz Pip-ε), 3.18 (3H, s, MeLeu-*N*-Me), 3.47 (1H, dd, J = 5.0, 9.0 H, MeLeu-α), 3.67 (1H, dd, J = 3.0, 11.5 Hz, Pip- α), 4.12 (1H, d, J = 13.5 Hz, Pip-ε), 4.30 (1H, dd, J = 3.0, 7.5 Hz, Val- α), 4.55 (1H, m, Leu- α), 4.80 (1H, m, Ala- α), 7.11 (1H, t, J = 7.5 Hz ABA-3), 7.18 (1H, d, J = 7.5 Hz, ABA-5), 7.45 (1H, t, J = 13.5 Hz, ABA-4), 7.62 (1H, d, J = 9.5 Hz, Ala-NH), 7.99 (1H, d, J = 8.0 Hz, Leu-NH), 8.28 (1H, d, J = 8.0 Hz, Val-NH), 9.39(1H, s, ABA-NH). ^{13}C-NMR (125 MHz, $CDCl_3$) δ: 16.2 (Val-γ), 18.4 (Ala-β), 19.8 (Ala-γ'), 21.8 (Leu-δ), 22.1 (MeLeu-δ)

23.2 (Leu-δ'), 23.3 (MeLeu-δ'), 24.4 (Leu-γ), 24.5 (Pip-β), 25.6 (MeLeu-γ), 27.4 (Pip-δ), 28.2 (Pip-γ), 29.9 (Val-δ) 36.3 (Leu-β), 37.8 (MeLeu-β), 37.9 (MeLeu-*N*-Me), 47.9 (Ala- α), 50.9 (Leu- α), 52.7 (Pip-ε), 59.2 (Val-α), 61.6 (Pip-α), 65.2 (MeLeu-α), 122.8 (ABA-1), 123.3 (ABA-3), 123.9 (ABA-2), 127.2 (ABA-5), 131.8 (ABA-4), 137.2 (ABA-6), 168.9 (Pip-CO), 169.3 (MeLeu-CO), 170.2 (ABA-CO), 170.7 (Val-CO), 174.2 (Leu-CO), 174.4 (Ala-CO).
